# Supplementary figures and images for: Expanded ACE2 dependencies of diverse SARS-like coronavirus receptor binding domains
Source: PLoS Biol. 2022 Jul 27;20(7):e3001738. doi: 10.1371/journal.pbio.3001738 (PMC9359572; doi:10.1371/journal.pbio.3001738)

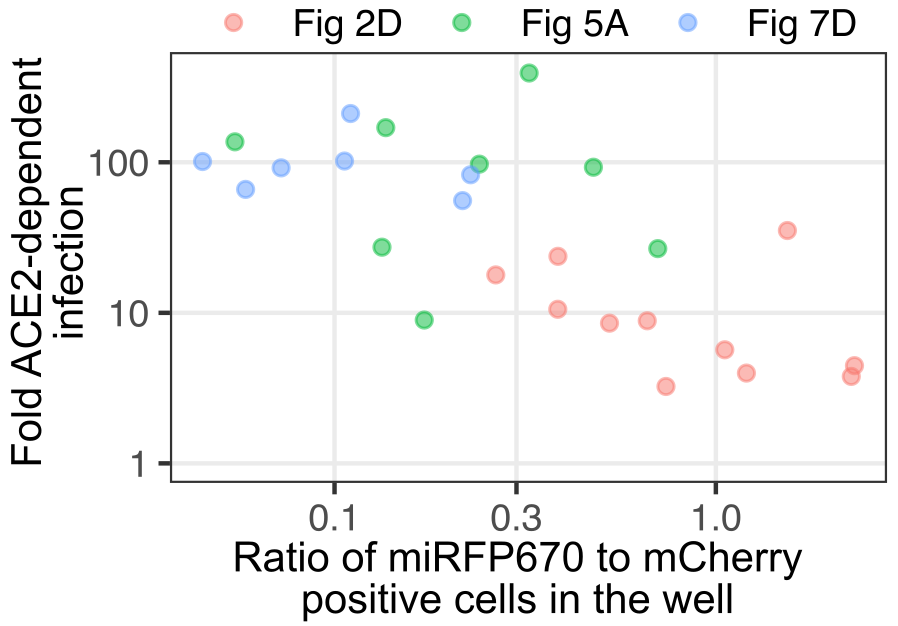

Supplement: S1 Fig — Scatter plot showing the calculated ratio of percent miRFP670 cells divided by percent mCherry-positive cells in the well for each SARS-CoV pseudovirus infection samples collected and analyzed for the indicated experiment (x-axis), compared to the fold ACE2-dependent infection observed for each of the samples (percent GFP-positive cells within the miRFP670-positive subset, divided by the percent GFP-positive cells within the mCherry-positive subset). The underlying data can be found in S2 Data, and the source code can be found at https://github.com/MatreyekLab/ACE2_dependence. ACE2, angiotensin converting enzyme-2; GFP, green fluorescent protein; SARS-CoV, Severe Acute Respiratory Syndrome-related Coronavirus. (TIF) [file pbio.3001738.s001.tif]

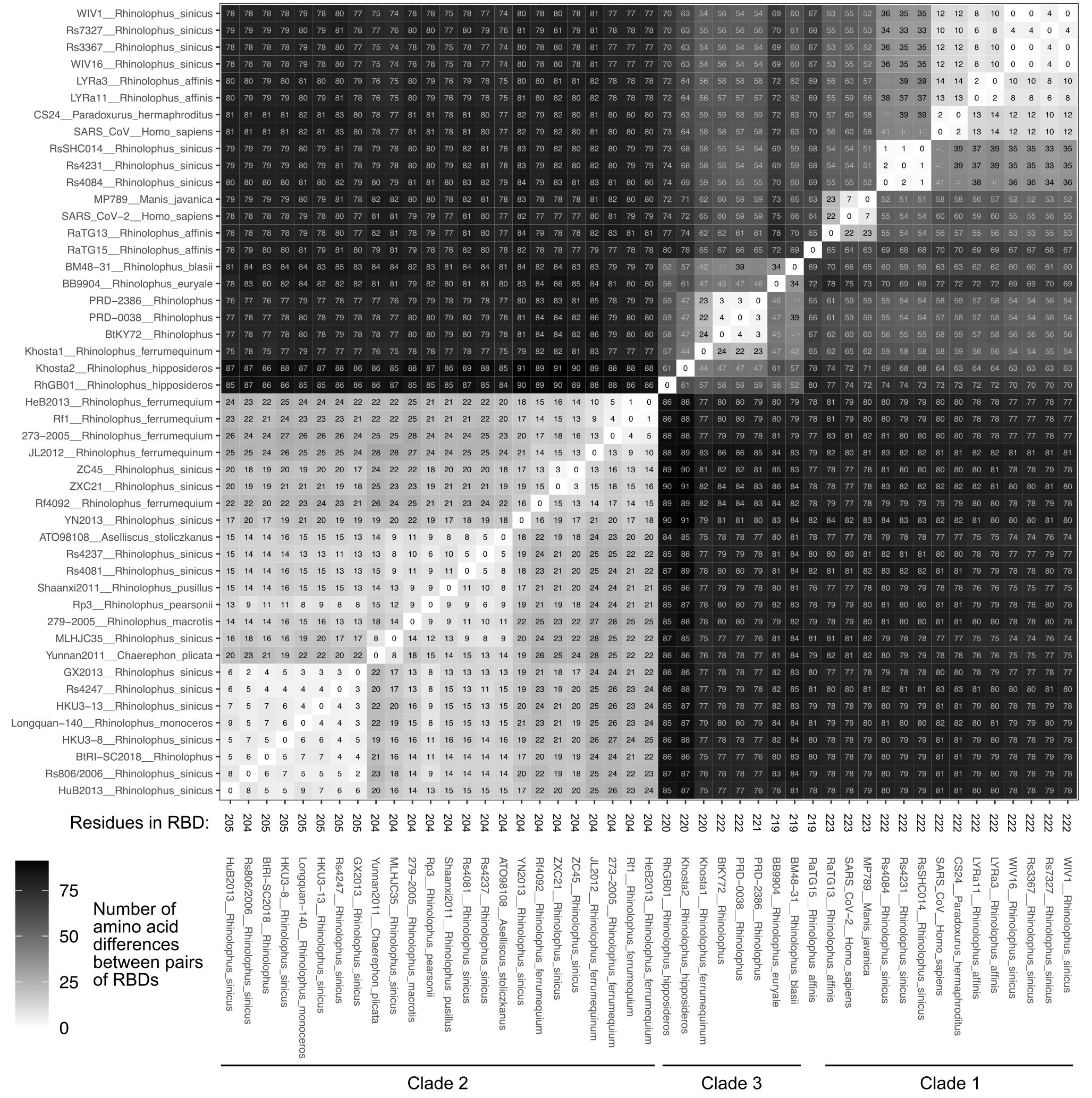

Supplement: S2 Fig — Similar to Fig 3E, except a larger set of RBDs were used as input. While not all known RBDs are shown, the smallest subset of samples capturing the known diversity of RBD sequences were chosen. The numbers within the boxes denote the number of residues that differ between the pairs of sequences. The numbers along the bottom axis labels denote the total number of residues in the RBD. The underlying data can be found in S2 Data, and the source code can be found at https://github.com/MatreyekLab/ACE2_dependence. RBD, receptor binding domain. (TIF) [file pbio.3001738.s002.tif]

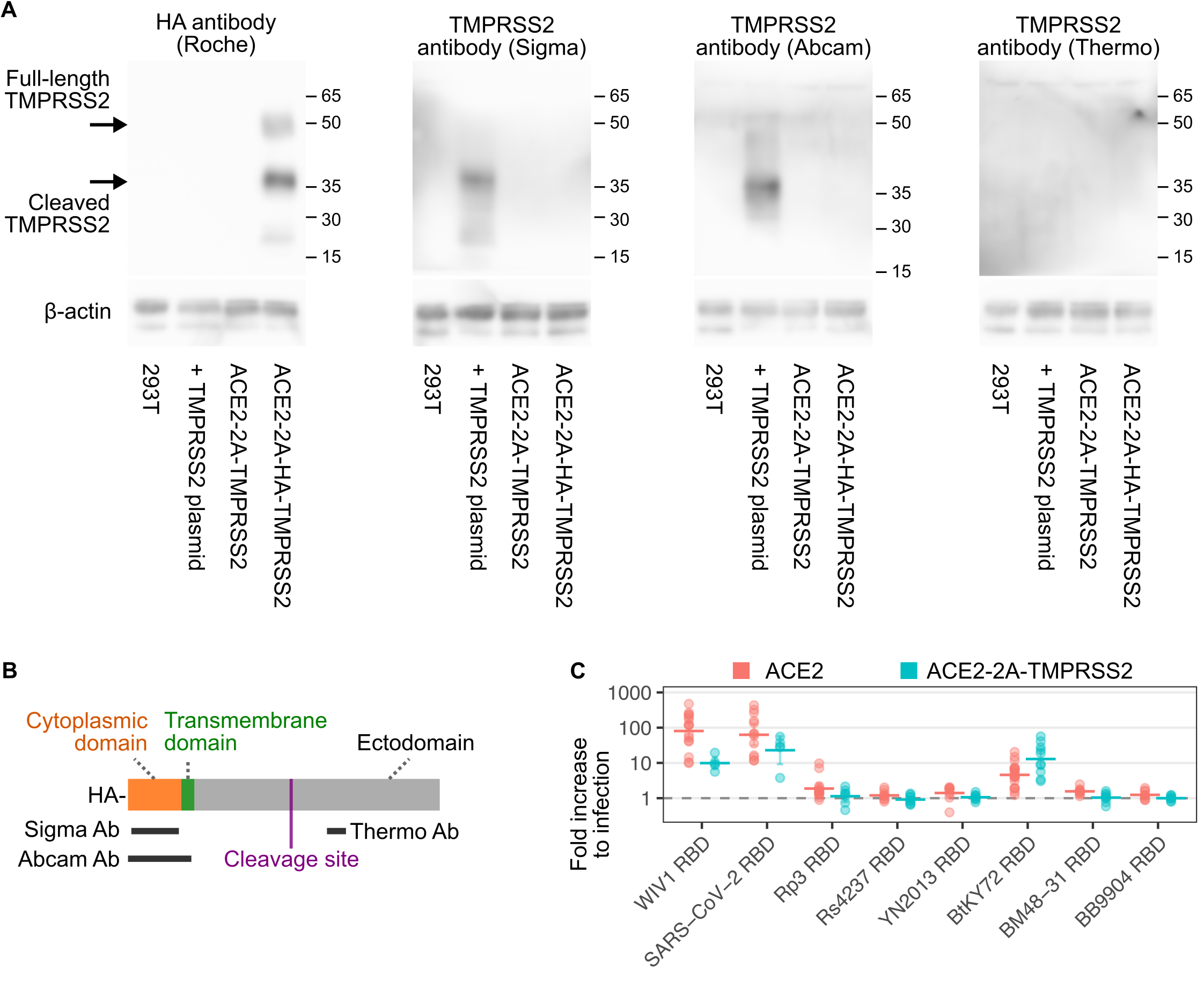

Supplement: S3 Fig — (A) Immunoblotting of lysate subjected to SDS polyacrylamide gel electrophoresis, collected from unmodified HEK 293T cells, HEK 293T cells transiently transfected with a plasmid encoding TMPRSS2 behind a CMV promoter, 293T landing pad cells stably recombined with a plasmid encoding ACE2 cotranslationally linked to either untagged or cytoplasmically HA-tagged TMPRSS2 using a 2A translational stop-start element. The top images show blots performed with an anti-HA antibody, or 3 different antibodies raised against the human TMPRSS2 protein sequence. The bottom images show beta-actin loading controls. (B) A schematic diagram showing the topological domains of TMPRSS2, its internal autoproteolytic cleavage site, and the peptide sequences used as immunogens during antibody creation. (C) Comparison of ACE2-dependent infectivities observed with HEK 293T cells overexpressing ACE2 only or ACE2 with TMPRSS2. The underlying data can be found in S2 Data, and the source code can be found at https://github.com/MatreyekLab/ACE2_dependence. ACE2, angiotensin converting enzyme-2; RBD, receptor binding domain; SARS-CoV-2, Severe Acute Respiratory Syndrome-related Coronavirus 2. (TIF) [file pbio.3001738.s003.tif]

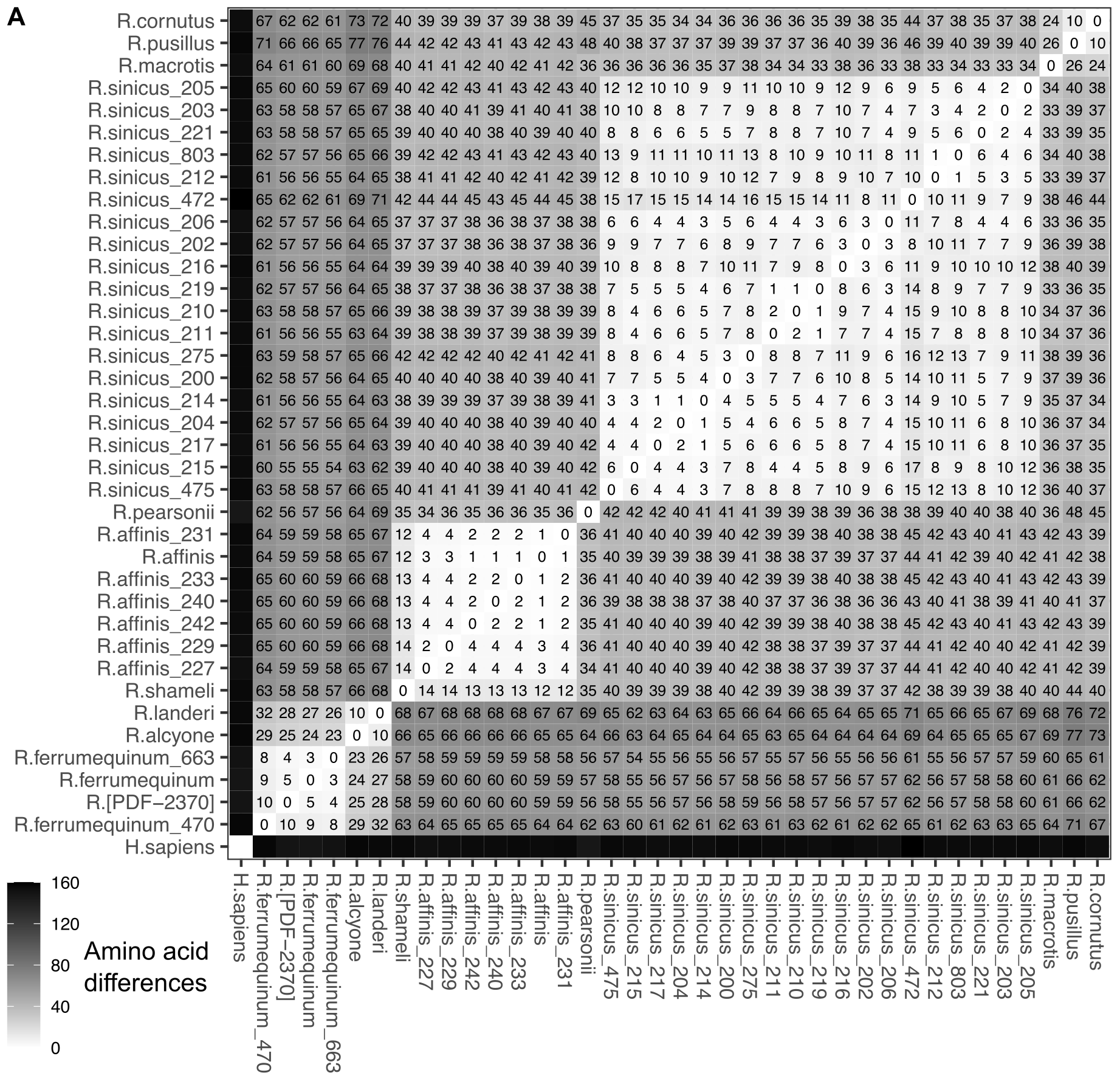

Supplement: S4 Fig — Unlike Fig 4C, which shows the amino acid differences in the chimeric ACE2 proteins we tested, this figure shows the Hamming distances for the full length protein sequences. The underlying data can be found in S2 Data, and the source code can be found at https://github.com/MatreyekLab/ACE2_dependence. ACE2, angiotensin converting enzyme-2. (TIF) [file pbio.3001738.s004.tif]

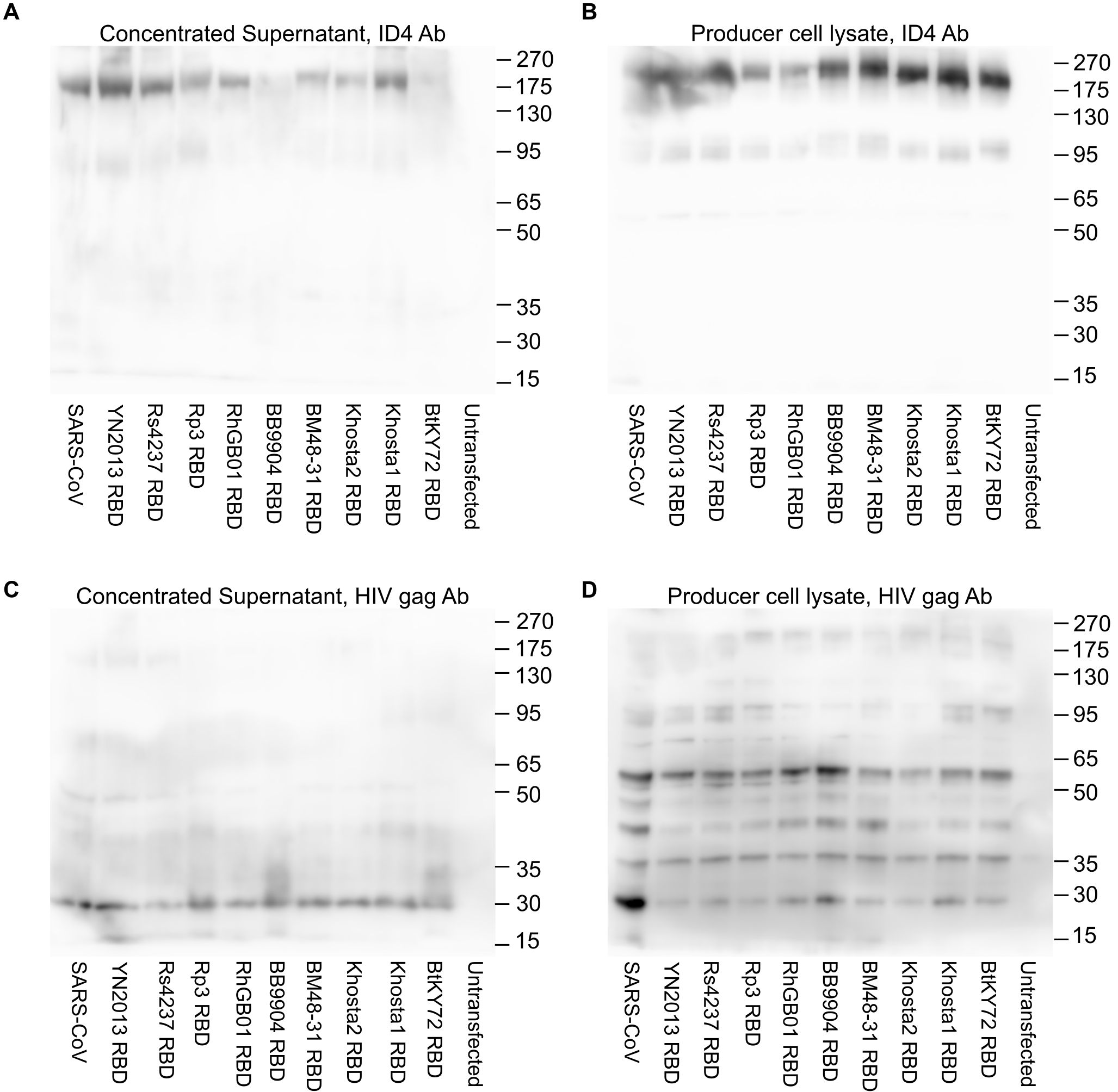

Supplement: S5 Fig — Lysates from concentrated virus preps are shown in panels A and C, while lysates collected from the corresponding producer cells are shown in panels B and D. Western blotting results using an antibody recognizing an epitope tag in the C-terminal end of the SARS-CoV cytoplasmic domain common to all constructs are shown in panels A and B, while results from incubation with a polyclonal antibody recognizing the HIV-1 structural polyprotein Gag is shown in panels C and D. The numbers to the right of each blot are the molecular weight of each band in the ladder, in kilodaltons. RBD, receptor binding domain; SARS-CoV, Severe Acute Respiratory Syndrome-related Coronavirus. (TIF) [file pbio.3001738.s005.tif]

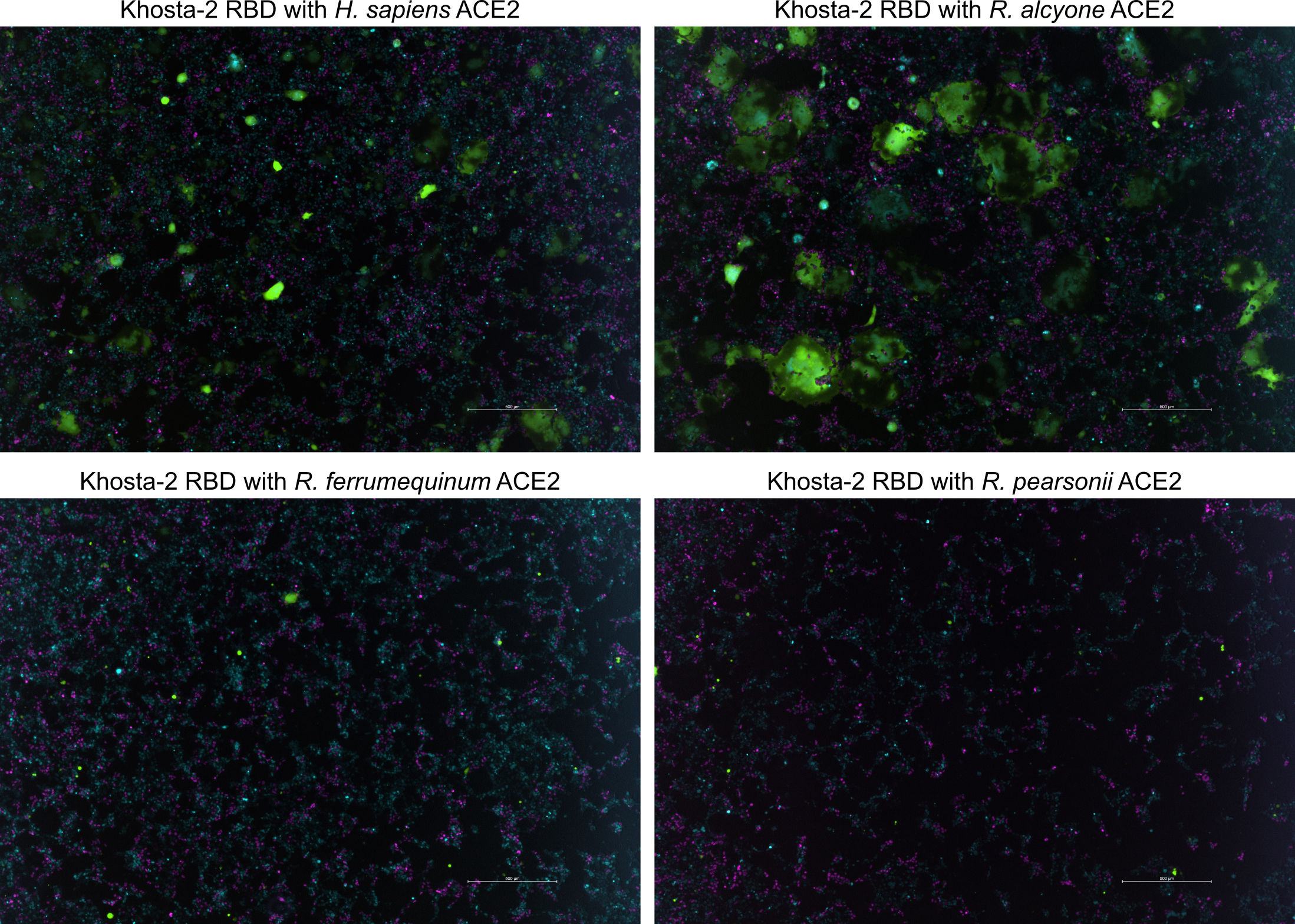

Supplement: S6 Fig — Green fluorescence marks cell bodies from syncytia in which at least 1 cell had been infected by Khosta-2 RBD pseudovirus. Magenta dots are ACE2-negative control cell nuclei expressing mCherry-fused histone H2A, while cyan dots are ACE2 ortholog cell nuclei expressing iRFP670-fused histone H2A. Images were taken with a 4× objective, and with a 500-μm scale bar shown at the bottom right of the image. All experiments were performed with HEK 293T cells overexpressing the indicated ACE2 sequence and human TMPRSS2 cotranslationally linked together with a 2A translational stop-start sequence. ACE2, angiotensin converting enzyme-2; RBD, receptor binding domain. (TIF) [file pbio.3001738.s006.tif]

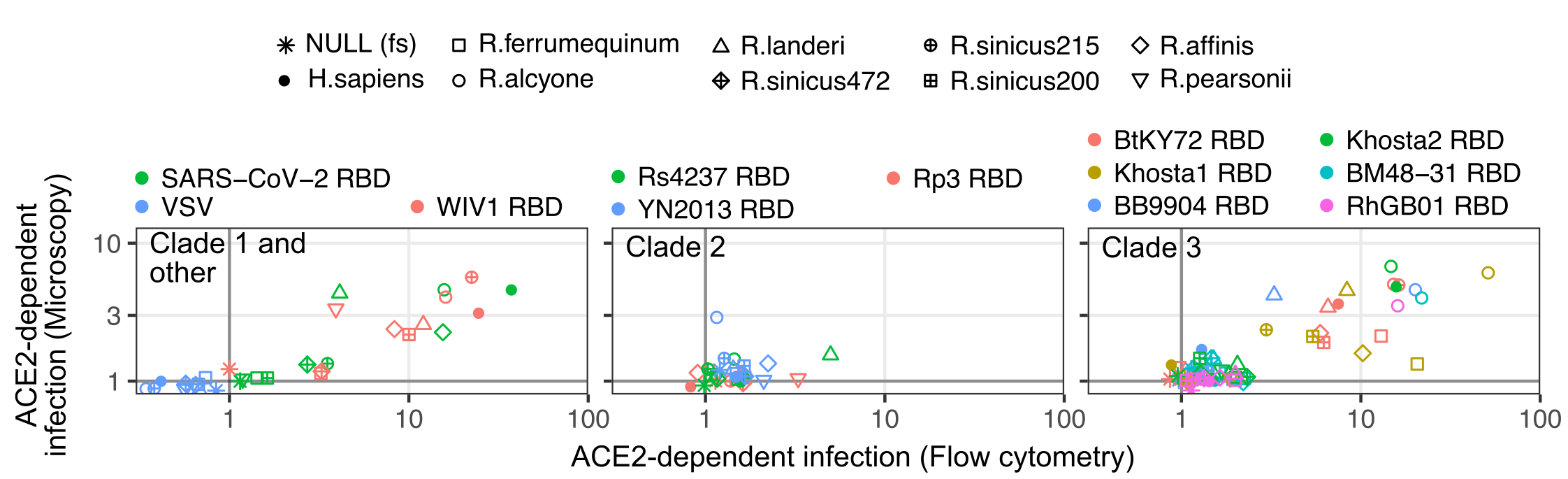

Supplement: S7 Fig — ACE2 dependence values calculated following flow cytometry values are shown on the left, while values calculated through microscopy are shown on the right. All experiments were performed with HEK 293T cells overexpressing the indicated ACE2 sequence and human TMPRSS2 cotranslationally linked together with a 2A translational stop-start sequence. The underlying data can be found in S2 Data, and the source code can be found at https://github.com/MatreyekLab/ACE2_dependence. ACE2, angiotensin converting enzyme-2; RBD, receptor binding domain; SARS-CoV-2, Severe Acute Respiratory Syndrome-related Coronavirus 2; VSV, vesicular stomatitis virus. (TIF) [file pbio.3001738.s007.tif]

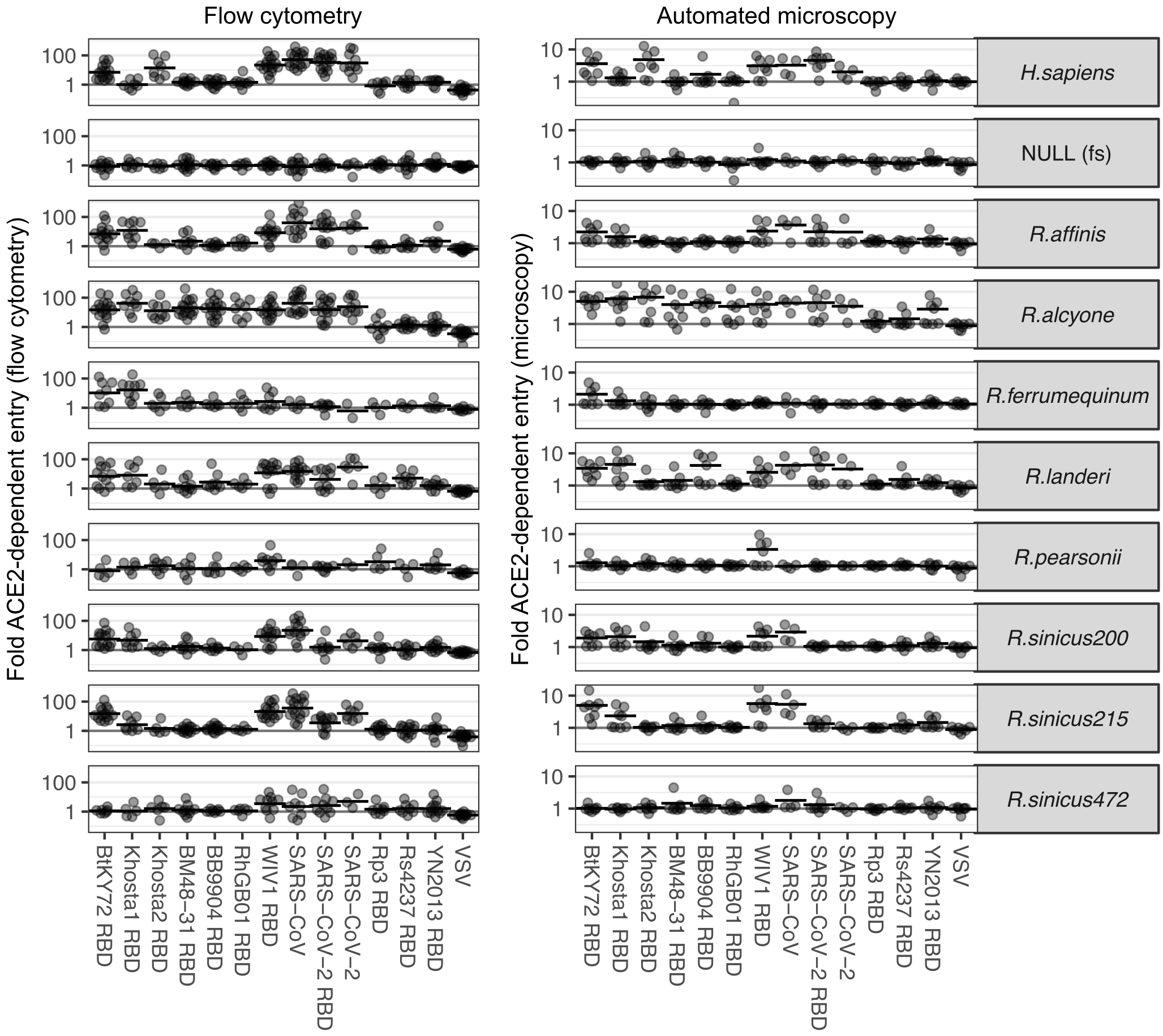

Supplement: S8 Fig — Scatter plots showing the correlation of ACE2-dependent infection quantitated with flow cytometry (x-axis) and microscopy (y-axis), separated by sarbecovirus clades. All experiments were performed with HEK 293T cells overexpressing both ACE2 and human TMPRSS2, linked together with a 2A translational stop-start element. The underlying data can be found in S2 Data, and the source code can be found at https://github.com/MatreyekLab/ACE2_dependence. ACE2, angiotensin converting enzyme-2; RBD, receptor binding domain; SARS-CoV, Severe Acute Respiratory Syndrome-related Coronavirus; SARS-CoV-2, Severe Acute Respiratory Syndrome-related Coronavirus 2; VSV, vesicular stomatitis virus. (TIF) [file pbio.3001738.s008.tif]

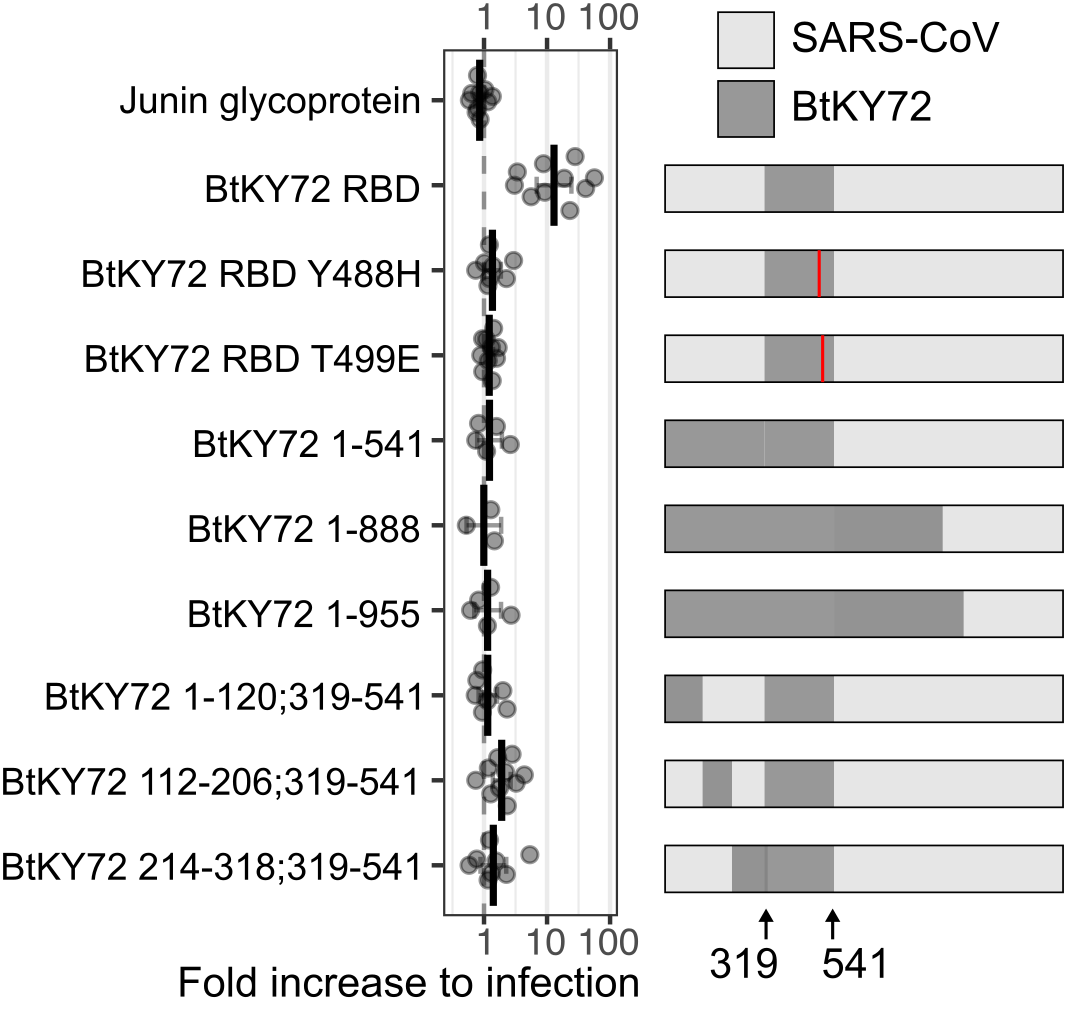

Supplement: S9 Fig — The schematic on the right shows the chimeric swap points that were tested with light gray denoting SARS-CoV sequence, and dark gray denoting BtKY72 sequence. The underlying data can be found in S2 Data, and the source code can be found at https://github.com/MatreyekLab/ACE2_dependence. ACE2, angiotensin converting enzyme-2; RBD, receptor binding domain; SARS-CoV, Severe Acute Respiratory Syndrome-related Coronavirus. (TIF) [file pbio.3001738.s009.tif]

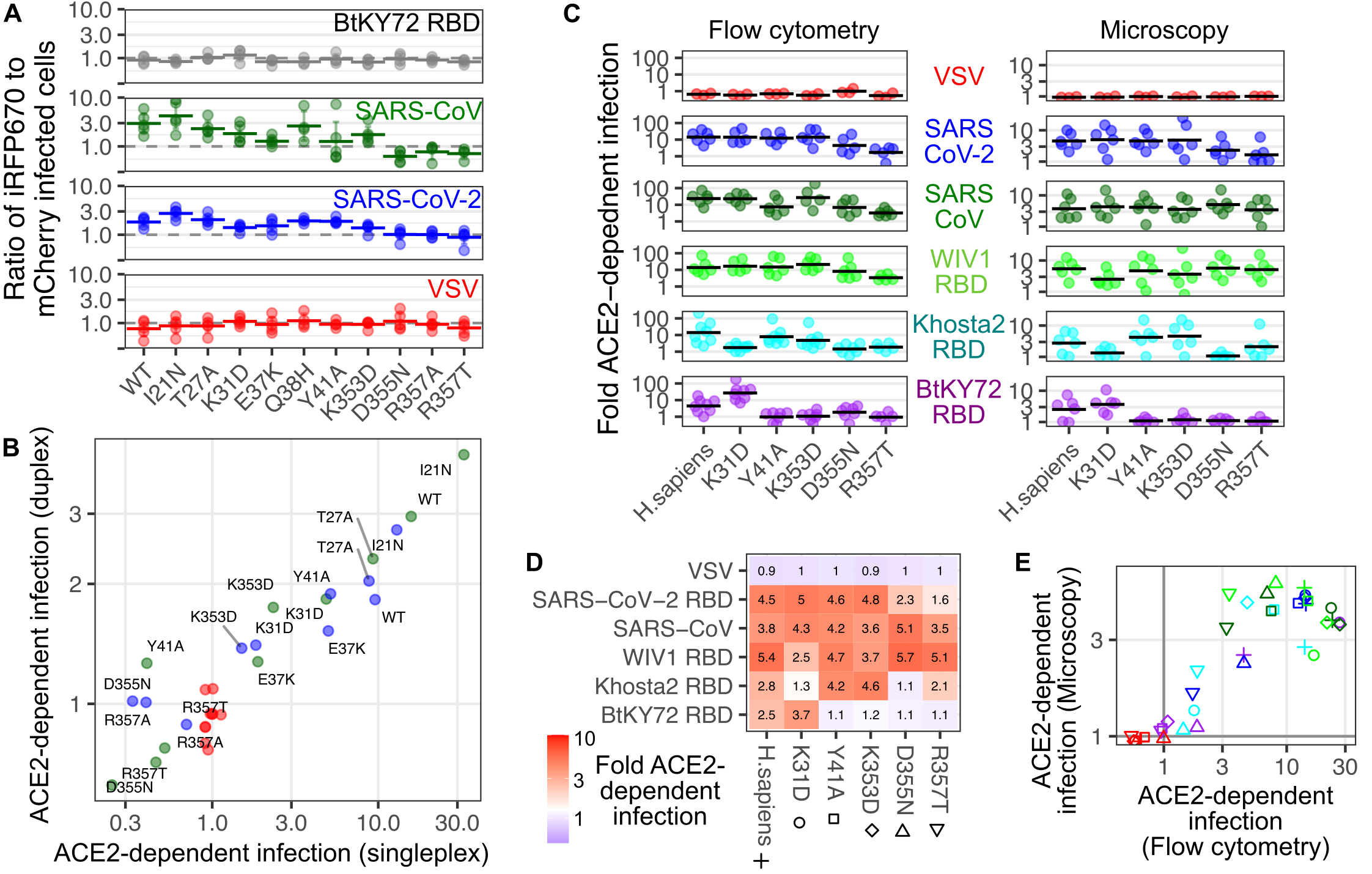

Supplement: S10 Fig — (A) ACE2-dependent infection of cells expressing WT or variant human ACE2 with chimeric RBD pseudoviruses, as determined by flow cytometry. These cells expressed relatively low cell surface ACE2 due to translation of the transgenic mRNA stimulated by a suboptimal Kozak sequence. (B) Correlation in ACE2-dependent infectivities as captured by the duplex assay shown in panel A compared with the results we previously obtained using the singleplex assay in a prior publication. (C) Flow cytometry and microscopy-based ACE2-dependent values observed with the WT or variant ACE2 proteins when encoded behind a consensus Kozak sequence and coexpressed with TMPRSS2. (D) Heatmap showing the ACE2-dependent values obtained through microscopy. (E) Correlation of geometric mean ACE2-dependent infectivities observed with the flow cytometry and microscopy readouts of the infection assay. Colors are as shown in panel C. Symbols are as labeled in panel D. The underlying data can be found in S2 Data, and the source code can be found at https://github.com/MatreyekLab/ACE2_dependence. ACE2, angiotensin converting enzyme-2; RBD, receptor binding domain; SARS-CoV, Severe Acute Respiratory Syndrome-related Coronavirus; SARS-CoV-2, Severe Acute Respiratory Syndrome-related Coronavirus 2; VSV, vesicular stomatitis virus; WT, wild-type. (TIF) [file pbio.3001738.s010.tif]

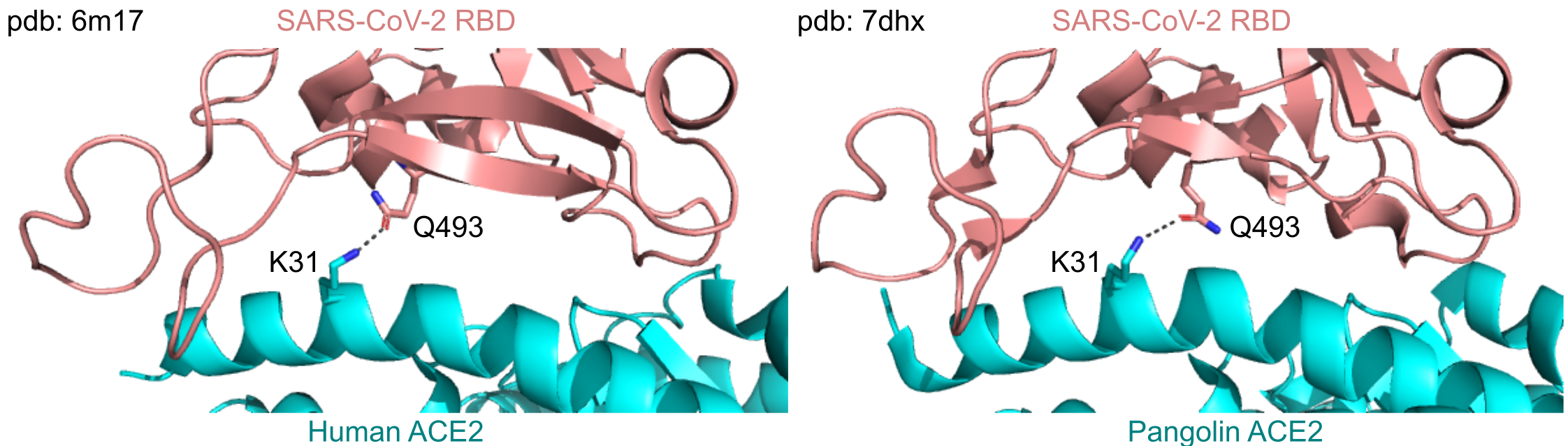

Supplement: S11 Fig — (A) A cryo-electron microscopy structure (PDB: 6m17, left) and an X-ray diffraction structure (PDB: 7dhx, right) between SARS-CoV-2 RBD and either human (left) or pangolin (right) ACE2. The side chain residues for ACE2 Lys31 and SARS-CoV-2 RBD Gln493 are shown as stick representations, with nitrogen atoms colored blue and oxygen atoms colored red. ACE2 is colored cyan, and SARS-CoV-2 RBD is colored salmon. The predicted hydrogen bond is shown as black dashes. ACE2, angiotensin converting enzyme-2; RBD, receptor binding domain; SARS-CoV-2, Severe Acute Respiratory Syndrome-related Coronavirus 2. (TIF) [file pbio.3001738.s011.tif]

Raw Images for Figure 2B

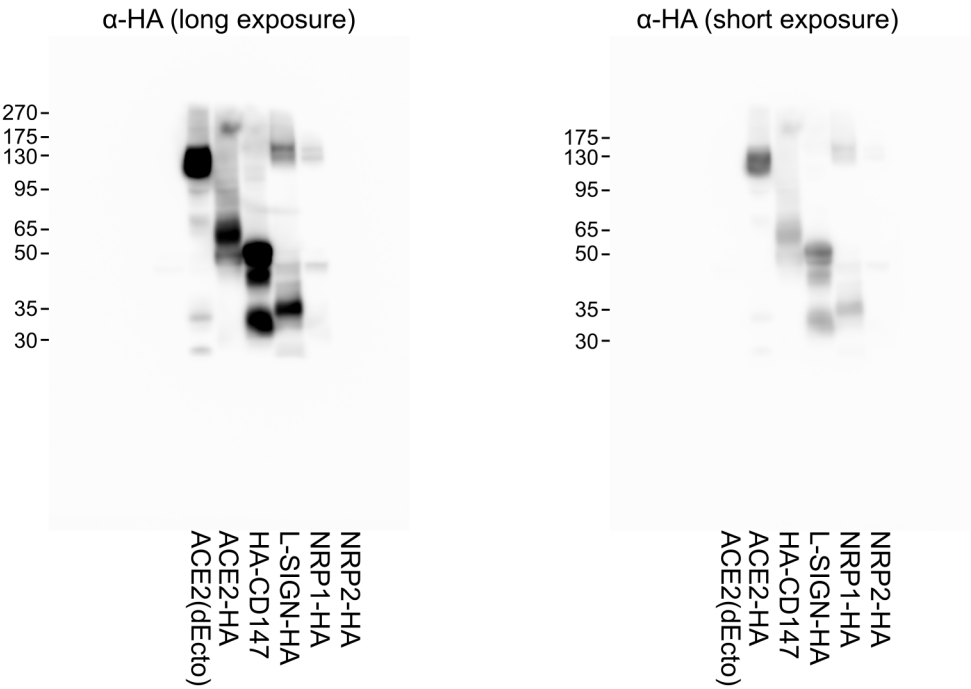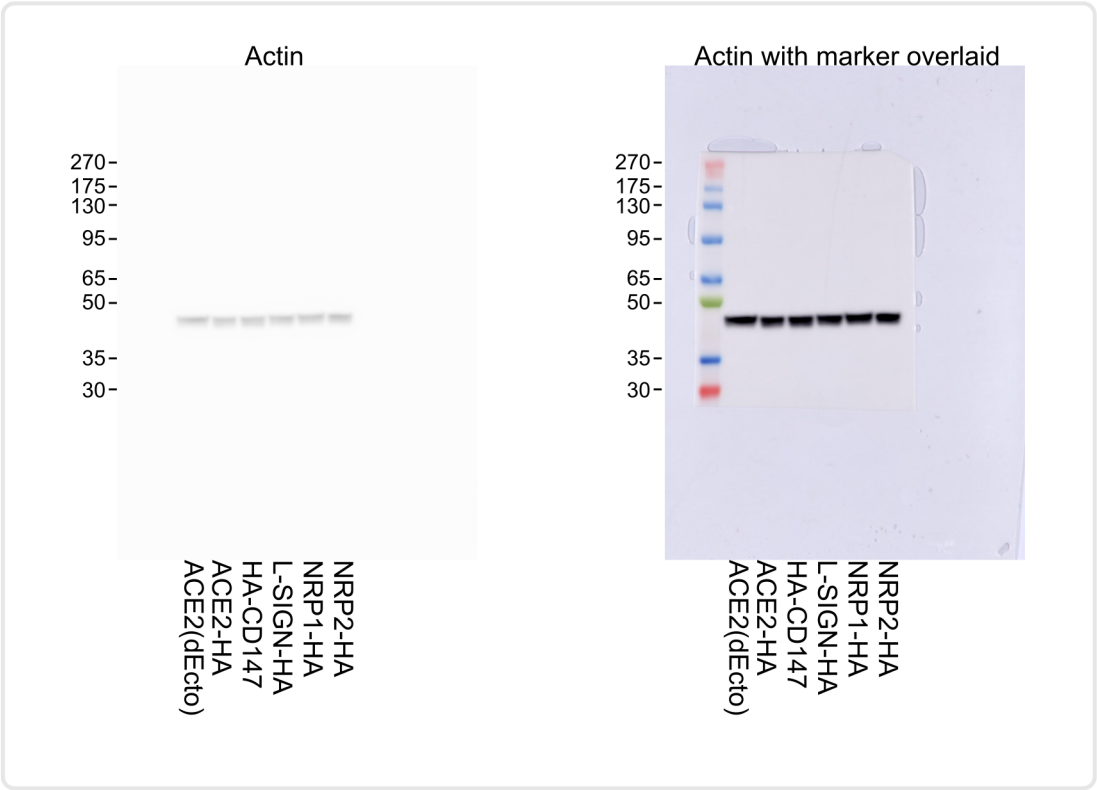

Raw Images for Figure 4D

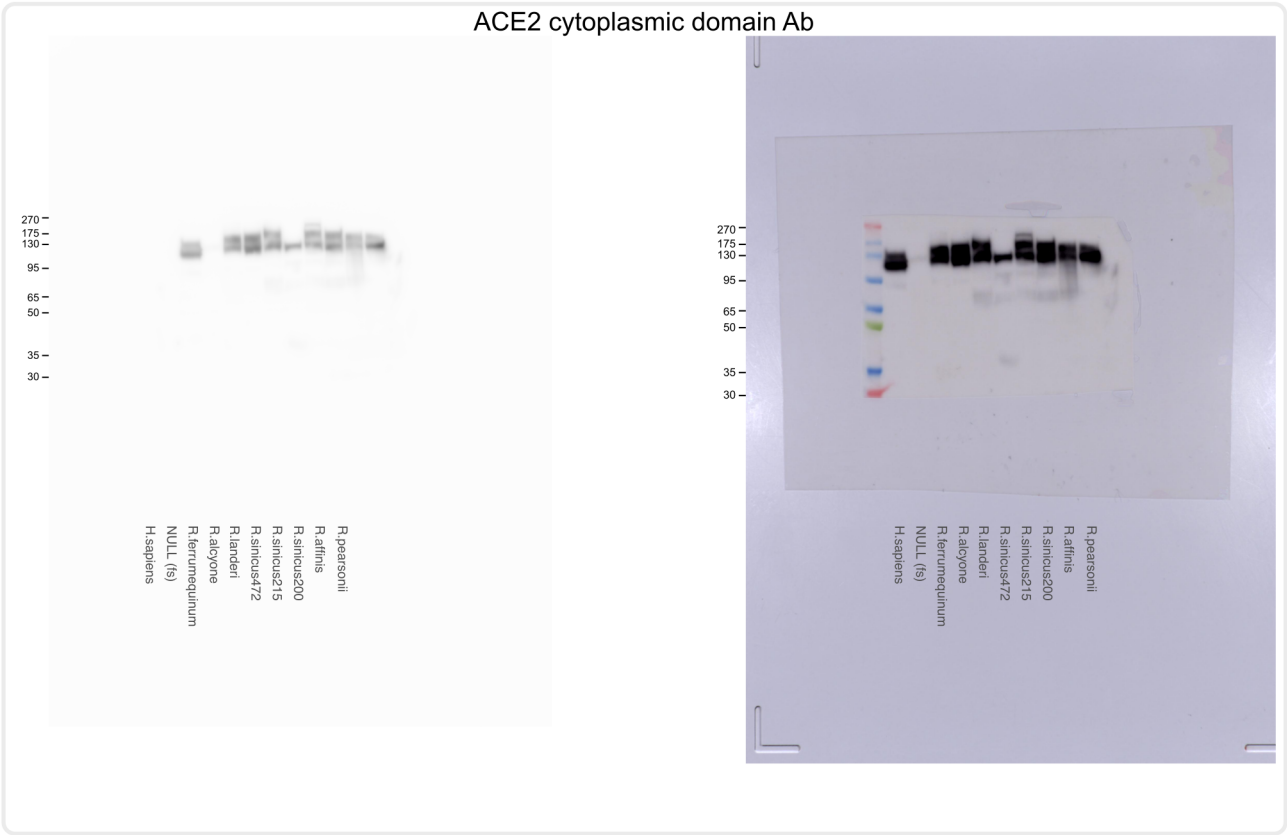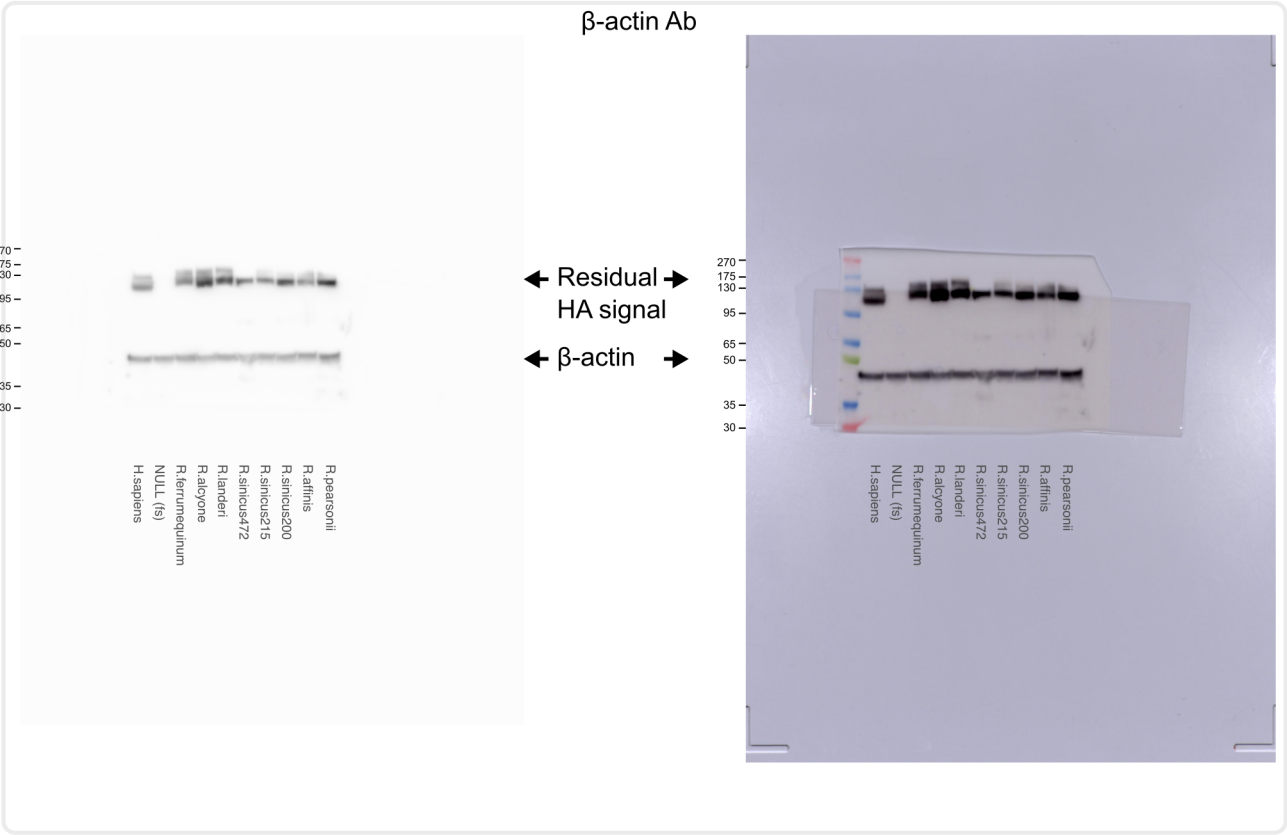

Raw Images for Figure SFig 3A

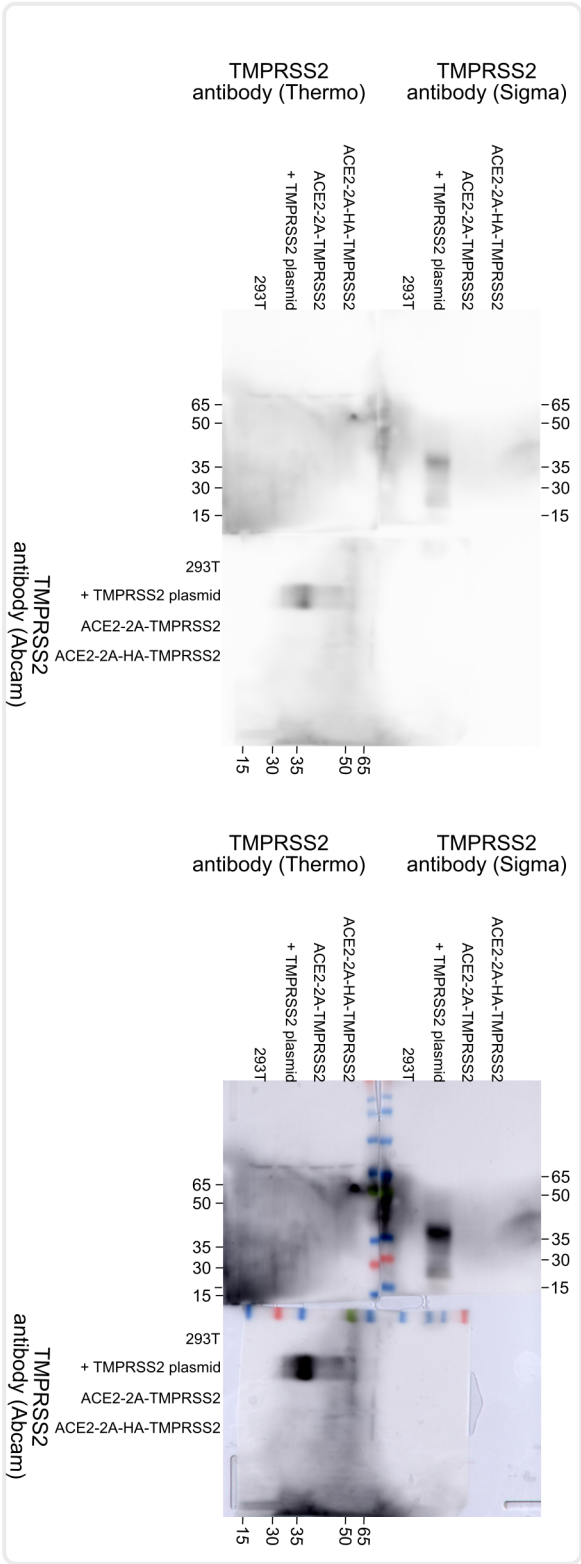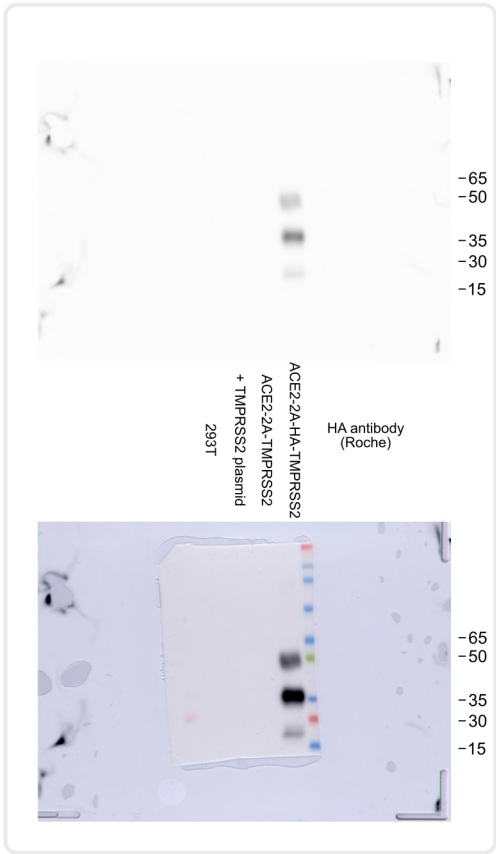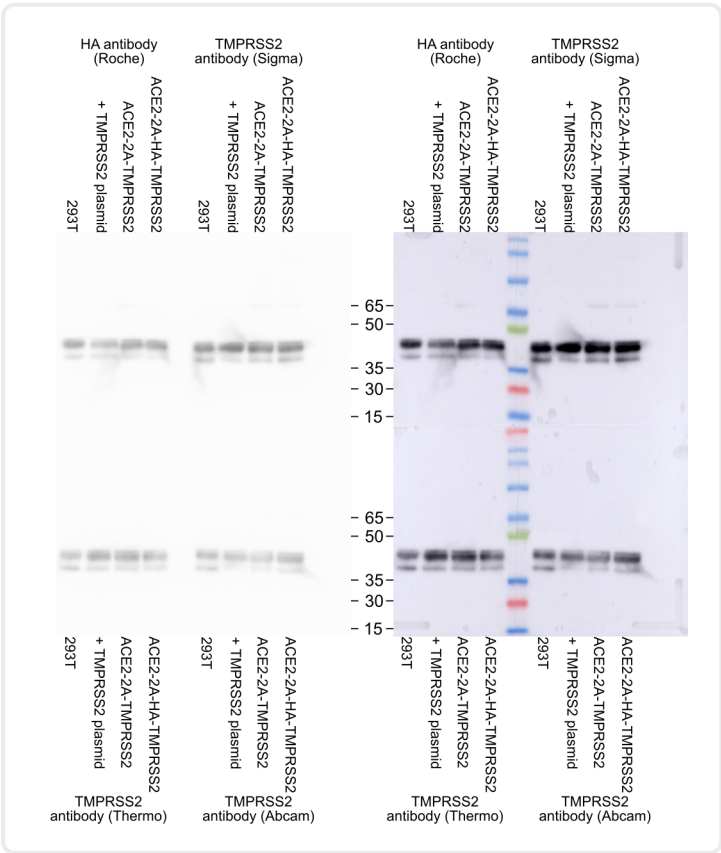

Raw Images for SFig 5

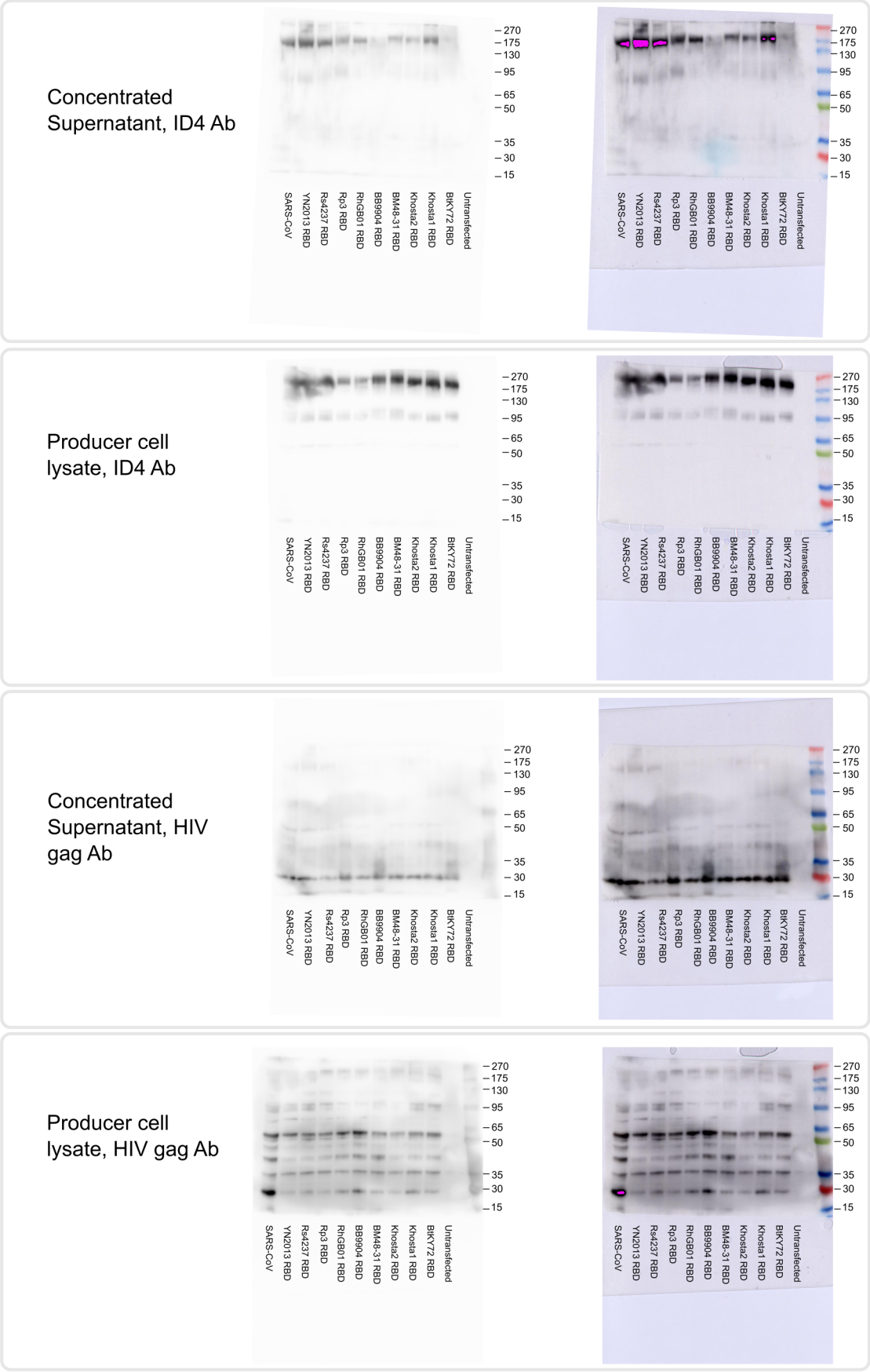

Supplement: S1 Raw Images — (PDF) [file pbio.3001738.s015.pdf]
